# Global Patterns and Temporal Trends in Ovarian and Uterine Cancer Mortality Attributable to High Body-Mass Index, 1990–2023

**DOI:** 10.3390/cancers18010157

**Published:** 2026-01-02

**Authors:** Irena Ilic, Vladimir Jakovljevic, Srdjan Lazic, Milena Ilic

**Affiliations:** 1 Faculty of Medicine, University of Belgrade, 11000 Belgrade, Serbia; 2 Department of Physiology, Faculty of Medical Sciences, University of Kragujevac, 34000 Kragujevac, Serbia; 3 Institute of Epidemiology, Military Medical Academy, 11000 Belgrade, Serbia; 4 Department of Epidemiology, Faculty of Medical Sciences, University of Kragujevac, 34000 Kragujevac, Serbia

**Keywords:** ovarian cancer, uterine cancer, high body-mass index, mortality, trends

## Abstract

**Background/Objectives**: The risk factors for ovarian and uterine cancer remain insufficiently known. This study aimed to assess global trends in mortality from ovarian and uterine cancer attributable to high body-mass index (BMI) in 1990–2023. **Methods**: An observational epidemiological study was conducted. The age-standardized rates (ASRs) of mortality were retrieved from the Global Burden of Disease study. Trends were evaluated using joinpoint analysis. The Average Annual Percentage Change (AAPC, %), with a 95% Confidence Interval (CI), was calculated. **Results**: Globally, the trend in ASRs of ovarian cancer deaths attributable to high BMI increased significantly (AAPC = +0.4%, 95% CI = 0.3 to 0.5). The growth trend in South Asia (AAPC = +8.7%, 95% CI = 8.1 to 9.2) was 30 times greater than in Eastern Europe (AAPC = +0.3%, 95% CI = 0.1 to 0.5). Declining trends in mortality from ovarian cancer were observed only in Australasia (AAPC = −0.2%, 95% CI = −0.4 to −0.1), High-income North America (AAPC = −0.3%, 95% CI = −0.6 to −0.0), and Western Europe (AAPC = −0.7%, 95% CI = −0.8 to −0.6). For uterine cancer, the global trend of mortality also increased (AAPC = +0.1, 95% CI = 0.0 to 0.2), with the trend growing fastest in South Asia (AAPC = +4.2%, 95% CI = 4.0 to 4.4). Decreasing trends in mortality from uterine cancer due to high BMI were observed only in Central Asia (AAPC = −0.6%, 95% CI = −0.9 to −0.4), East Asia (AAPC = −2.2%, 95% CI = −2.6 to −1.8), and Southern Latin America (AAPC = −0.4%, 95% CI = −0.6 to −0.1). Additionally, an accelerated increase in mortality trends for both ovarian and uterine cancer attributable to high BMI was observed in 2020–2023, which corresponds to the period of the COVID-19 pandemic. **Conclusions**: Further analytical epidemiological studies are required to clarify the relationship between ovarian and uterine cancer and high BMI.

## Simple Summary

Although several risk factors for ovarian and uterine cancers are well-documented, these diseases still represent major public health concerns for women worldwide, and the full complexity of the drivers of global mortality trends requires further clarification. The main goal of this study was to evaluate global mortality of ovarian and uterine cancer attributable to high body-mass index (BMI) in 1990–2023. A total of 7.7 million deaths from ovarian and uterine cancer together were recorded worldwide (5.1 million ovarian cancer deaths and 2.6 million uterine cancer deaths) in the period observed. Globally, the fraction of deaths from ovarian cancer attributable to high BMI in 2023 was 8.65%, whereas for uterine cancer it reached 33.33%. From 1990 to 2023, global trends in mortality from both ovarian and uterine cancer attributable to high BMI increased significantly, with an accelerated growth during 2020–2023, a period which corresponds to the COVID-19 pandemic.

## 1. Introduction

According to 2022 estimates from the World Health Organization (WHO), ovarian cancer ranks as the 7th most frequently diagnosed malignancy, accounting for 3.4% of all female cancers in the world; it is also the 6th leading cause of cancer-related death, representing 4.8% of all female cancer deaths [1]. In 12 countries, including India, Pakistan, Indonesia, and Sudan, ovarian cancer is the 3rd most common cancer among females of all ages [1]. Moreover, in 20 countries across Asia (e.g., India, Indonesia, Pakistan, Malaysia, Sri Lanka), Europe (e.g., Ukraine, Georgia), and Africa (e.g., Sudan, Niger), the WHO estimates ovarian cancer as the 3rd leading cause of cancer-related death in females in 2022 [1]. Ovarian cancer is the most fatal type of gynecological cancer; while the 5-year relative survival for localized-stage disease is about 90.0%, the overall survival rate is approximately 50% [1,2,3].

At the same time, uterine cancer was the 6th most commonly diagnosed malignancy worldwide, accounting for more than 4.3% of all female cancers, and ranked as the 13th leading cause of cancer-related death, responsible for 2.2% of all female cancer deaths [1]. In four Caribbean countries (Jamaica, Trinidad and Tobago, the Bahamas, and Barbados) uterine cancer is the 2nd most common cancer among females of all ages and the 3rd most common cancer in females in countries in Eastern Europe (including Russian Federation, Ukraine, Belarus, Bulgaria, Slovakia, Lithuania, Latvia, Armenia, Moldova, Estonia) [1]. Moreover, uterine cancer was ranked as the 4th to 5th leading cause of cancer-related death in females in a number of Caribbean countries (such as Cuba, Jamaica, Barbados, Trinidad and Tobago, and the Bahamas) in 2022 [1]. The 5-year overall survival rates are approximately 70–80% across an age-, gender-, site-, stage-, histological type-, and socioeconomic status-related gradient [2,4]

In 2022, the global age-standardized rate (ASR) of incidence for ovarian cancer was 6.7 per 100,000 women, with the highest rate in the WHO Region of Europe (8.9 per 100,000) and the lowest rate in the WHO Region of Africa (5.0 per 100,000) [1]. The global ASR of mortality from ovarian cancer was 4.0 per 100,000 women, with the highest rate in the WHO Region of Europe (5.0 per 100,000) and the lowest rate in the WHO Western Pacific Region (2.9 per 100,000) in 2022.

The global ASR of incidence for uterine cancer was 8.4 per 100,000 women in 2022, with the highest rate in the WHO Region of Europe (14.9 per 100,000) and the lowest rate in the WHO Region of South-East Asia (3.1 per 100,000) [1]. The global ASR of mortality from uterine cancer was 1.7 per 100,000 women in 2022, with the highest rate in the WHO Region of Europe (2.8 per 100,000) and the lowest rate in the WHO South-East Asia Region (1.1 per 100,000).

Most deaths from ovarian and uterine cancer worldwide occur in older age, predominantly among postmenopausal women [1,5,6]. In contrast to developed countries, where the majority of newly diagnosed cases of ovarian and uterine cancer occur in the oldest age groups, developing countries (the Republic of Korea, China, and Lithuania) exhibit a plateau in incidence during middle age (at the age of 50–60 years) with a decline in incidence in the oldest age [1,7].

Substantial heterogeneity in incidence and mortality trends for both ovarian and uterine cancer has been observed across populations worldwide since the mid-20th century [1,8,9]. In 2015, the WHO and the United Nations established the Sustainable Development Goals, which include reducing the risk of premature mortality from non-communicable diseases (including cancer) by one third by 2030 (indicator 3.4.1) around the world [10]. However, some studies have reported an increased incidence of endometrial cancer and malignant ovarian tumors across all clinical stages during the coronavirus disease 2019 (COVID-19) pandemic, indicating that the COVID-19 pandemic influenced patients to postpone visits to the doctor, leading to delays in diagnosis [11,12]. Furthermore, recent projections indicate that the global burden of ovarian and uterine cancer incidence will continue to rise over the next 30–40 years, driven by population aging, population growth, and the increasing prevalence of risk factors [8,13,14].

Aside from older age, previous studies have reported conflicting results regarding the role of various factors in the etiology of ovarian and uterine cancer (e.g., genetic factors, family history, personal medical history, drug use, reproductive factors, smoking, social and economic factors, diet, etc.) [15,16,17,18,19,20,21]. The relationship between obesity and ovarian cancer risk is inconsistent: whereas some studies have demonstrated a positive relationship between ovarian cancer and obesity [22,23], others have not found any relationship [24,25]. Contrary to this, obesity is more strongly positively associated with the development of uterine cancer than with any other gynecological cancer types [26,27,28]. Nevertheless, an improved understanding of the relationship between obesity and the burden of ovarian and uterine cancer could increase awareness among women of the general population and emphasize the role of obesity in cancer risk, prevention, and treatment. Accordingly, this manuscript aimed to investigate global patterns and temporal trends in mortality from ovarian and uterine cancer attributable to high body-mass index (BMI) over the last few decades.

## 2. Materials and Methods

### 2.1. Study Design

In order to explore the global patterns in temporal trends in mortality from ovarian and uterine cancer attributable to high BMI in 1990–2023, an observational descriptive epidemiological study, with an ecological study design, was conducted.

### 2.2. Data Sources

Data on the incidence and mortality from ovarian and uterine cancer were obtained from the Global Burden of Disease (GBD) 2023 study database [29]. The GBD database provides comprehensive, comparable, and reliable estimates of incidence and mortality for ovarian and uterine cancer. The 2023 iteration of the study encompasses up-to-date data for 204 countries and territories, covering 292 causes of death, and quantifying risk-attributable burden for 88 risk factors. The GBD estimates are compiled from an extensive array of data sources, including vital registration systems, cancer registries, verbal autopsy records, census data, national health surveys, research studies, and government reports. By integrating these data and employing rigorous statistical modeling, the GBD study produces high-quality, consistent estimates of ovarian and uterine cancer burden globally, adhering to the Guidelines for Accurate and Transparent Reporting of Health Estimates [30].

Estimates for ovarian cancer were determined using the 10th revision of the International Classification of Diseases and Related Health Problems to classify cause of disease and death (ICD-10) site codes C56-C56.9, D27-D27.9, and D39.1 [31,32], and malignant neoplasms as defined by the 9th revision of the ICD codes 183, 183.0, 220, 220.0, 220.9, 236.2 [31,33]. For uterine cancer, estimates encompassed site codes C54-C54.9, D07.0, D26.1, D26.7, and D26.9, based on the ICD-10 [30,31], and malignant neoplasms as defined by the ICD-9 codes 182, 182.0, 182.1, 182.8, 233.2 [31,33].

Data related to human development indicators—including Gross Domestic Product (GDP), GDP per capita, and the Human Development Index (HDI)—were obtained from the United Nations Statistics Division National Accounts Main Aggregates Database [34]. In addition, information regarding the Socio-development Index (SDI) was retrieved from the GBD study [29].

### 2.3. Variables and Measures

Age-specific and age-standardized rates (expressed per 100,000 persons) were calculated for this study. The direct standardization method was employed using the GBD standard population as the reference population to produce age-standardized rates (ASRs) [35].

The current study presents patterns and trends in mortality from ovarian and uterine cancer on the global, regional (by 21 GBD regions), and national level (for 204 countries and territories). Due to the resulting volatile rate estimates, the results are presented but not taken into account in the comparison for countries/territories with fewer than 100,000 inhabitants (such as Andorra, American Samoa, Antigua and Barbuda, Cook Islands, Dominica, Greenland, Marshall Islands, Monaco, Nauru, Niue, Northern Mariana Islands, Palau, Saint Kitts and Nevis, San Marino, Tokelau, and Tuvalu).

The GBD comparative risk assessment (CRA) framework is developed to estimate the proportion of disease burden attributable to a given risk factor by comparing observed population exposure to a theoretical minimum risk exposure level (TMREL) [36]. Attributable burden is quantified using population attributable fractions, representing the proportion of disease burden that might be avoided if exposure to a risk factor is reduced to the TMREL. These estimates rely on systematic reviews of the literature and meta-regression techniques used to synthesize relative risks while adjusting for study-level differences and heterogeneity. In the GBD CRA, high body-mass index is recognized as a risk factor for ovarian and uterine cancer, with the TMREL defined as a BMI of 20–25 kg/m^2^; attributable burden reflects exposure above this range, commonly referred to as high BMI > 25 kg/m^2^.

The Socio-development Index (SDI) is a composite measure of three indicators: income per capita, average years of schooling in those aged 15 and older, and total fertility rate for females under the age of 25 [29].

The Human Development Index (HDI) serves as a composite metric designed to evaluate human progress across three dimensions—health (represented by life expectancy), education (reflected through literacy and school enrollment), and standard of living (measured by income) [34]. Both SDI and HDI values are quantified on a scale ranging from 0 to 1, with higher values denoting greater development of a country.

### 2.4. Statistical Analysis

Temporal trends in mortality from ovarian and uterine cancer were examined using Joinpoint regression analysis software (Version 4.9.0.0; National Cancer Institute, Bethesda, MD, USA—March 2021), following the methodology proposed by Kim et al. [37]. The joinpoint regression analysis evaluates the magnitude and direction of trends by detecting specific temporal points at which there was a significant change in the trend (termed “joinpoints”). The Monte Carlo permutation test was used for multiple comparisons (at 4499 randomly selected datasets) [37]. The Grid Search Method was used [38]. For each model, a minimum of zero joinpoints (one line segment) and a maximum of five joinpoints (six line segments) were allowed. The value that describes the linear trend within each segment is the Annual Percent Change (APC), with its corresponding 95% Confidence Interval (CI). As an overall measure of trend over the entire considered period, the Average Annual Percent Change (AAPC) with the corresponding 95% CI was calculated [39]. Temporal trends were described using the term “significant change” (increase or decrease), indicating that the slope of the trend was statistically significant (*p* < 0.05), and as “stable” when they had an APC/AAPC whose confidence interval (CI) overlapped zero. Trends were not evaluated separately for persons aged <20 years due to the absence of ovarian and uterine cancer deaths in this age group. Disparities in trends of ovarian and uterine cancer mortality attributable to high BMI by age group were evaluated using the comparability test (test of parallelism) [40].

Additionally, the association between the age-standardized rates (ASRs) of mortality from ovarian and uterine cancer attributable to high BMI and measures of socioeconomic development was evaluated using linear regression models. Also, the correlation between the ASRs of mortality from ovarian and uterine cancer attributable to high BMI and measures of socioeconomic development was quantified using the Pearson Correlation coefficient. Statistical significance was accepted at the level of *p* < 0.05; analyses were performed using the SPSS Software (version 20.0, Chicago, IL, USA).

### 2.5. Ethical Considerations

The study was conducted according to the guidelines of the Declaration of Helsinki, and it was approved by the Ethics Committee of the Faculty of Medical Sciences, University of Kragujevac (Ref. No.: No. 01-14321), on 30/11/2017. This study was conducted using aggregated and non-identifiable data from publicly available sources.

## 3. Results

**Figure 1:**
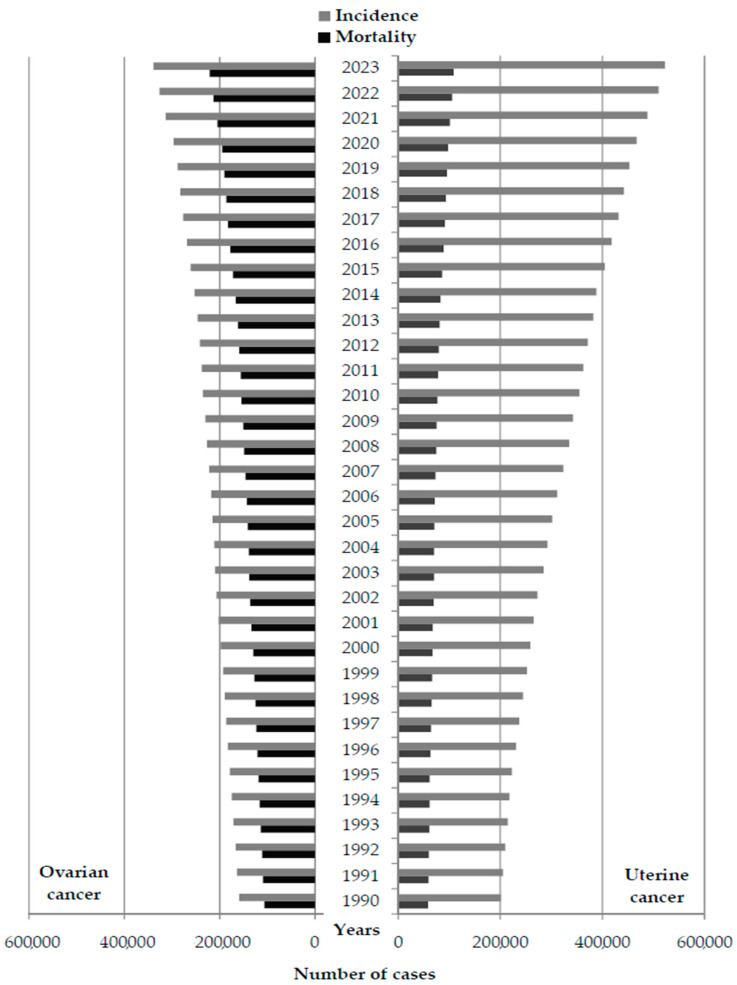
Number of new cases and deaths from ovarian and uterine cancer in the world, 1990–2023. Data source: Global Burden of Disease study [29].

A total of 19 million new cases of ovarian and uterine cancer combined were reported worldwide over the period 1990–2023 (7.8 million new cases of ovarian and 11.2 million new cases of uterine cancer); per year, the number of new cases ranged from 158,603 in 1990 to 338,615 in 2023 for ovarian cancer, and from 200,986 in 1990 to 522,661 in 2023 for uterine cancer (Figure 1). Over the same period, a total of 7.7 million deaths from ovarian and uterine cancer together were reported worldwide (5.1 million deaths from ovarian and 2.6 million deaths from uterine cancer); per year, the number of deaths ranged from 106,301 in 1990 to 220,979 in 2023 for ovarian cancer, and from 58,401 in 1990 to 108,592 in 2023 for uterine cancer.

**Figure 2:**
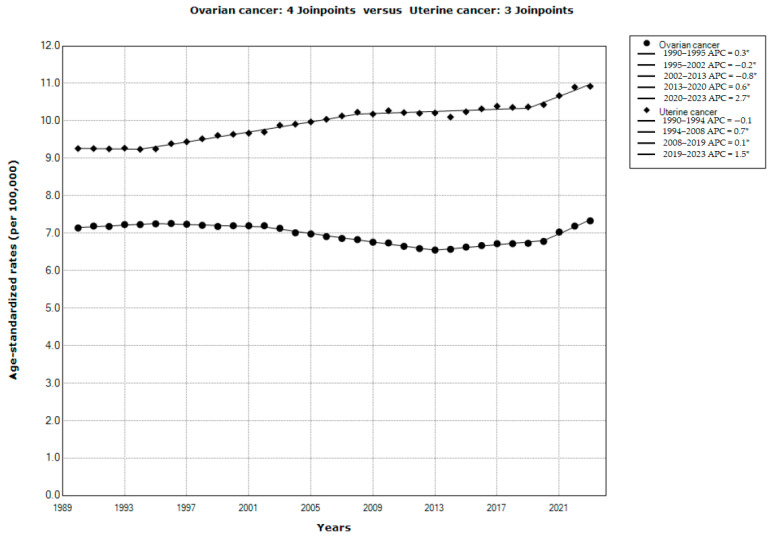
Joinpoint regression analysis of global trends in age-standardized rates of incidence of ovarian and uterine cancer, 1990–2023. * Statistically significant trend (*p* < 0.05). APC—annual percentage change. Data source: Global Burden of Disease study [29].

The results of the joinpoint regression analysis of the global trends in ovarian and uterine cancer incidence in the studied period, 1990–2023, are presented in Figure 2. A significant increase in incidence was observed both for uterine cancer (AAPC = +0.5%, 95% CI = 0.4 to 0.6) and for ovarian cancer (AAPC = +0.1%, 95% CI = 0.0 to 0.2). According to the comparability test, incidence trends for ovarian cancer and for uterine cancer were not parallel (Final selected model rejected parallelism, *p* < 0.001). There were four joinpoints in the incidence trend for ovarian cancer: incidence increased by +0.3% per year from 1990 to 1995, then significantly decreased (by −0.2% per year from 1995 to 2002, and by −0.8% per year from 2002 to 2013), followed by a significant increase by +0.6% per year from 2013 to 2020 and with an accelerated growth by +2.7% per year from 2020 until the end of the period in 2023. There were three joinpoints in the incidence trend for uterine cancer: incidence was stable (by −0.1% per year) from 1990 to 1994, followed by a significant increase from 1994 until the end of the period in 2023 (that is, by +0.7% per year from 1994 to 2008, by +0.1% per year from 2008 to 2019, and with accelerated growth by +1.5% per year from 2019 until the end of the period in 2023).

**Figure 3:**
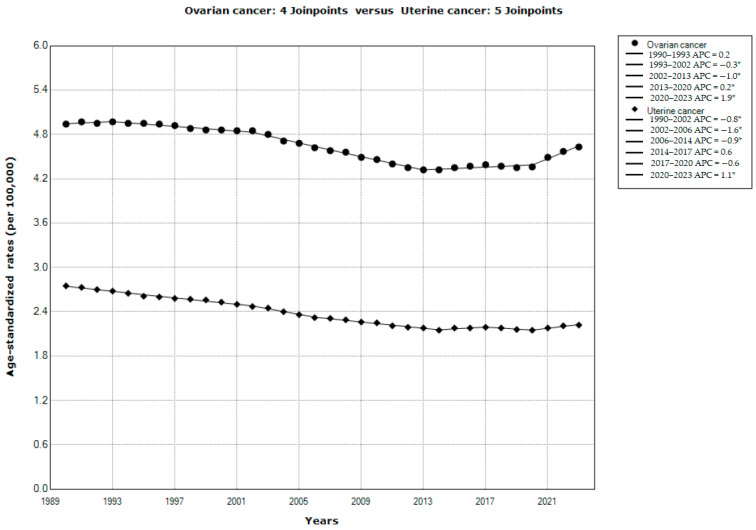
Joinpoint regression analysis of global trends in age-standardized rates of mortality from ovarian and uterine cancer, 1990–2023. * Statistically significant trend (*p* < 0.05). APC—annual percentage change. Data source: Global Burden of Disease study [29].

Results of the joinpoint regression analysis of the global trends in mortality of ovarian and uterine cancer in the studied period 1990–2023 are presented in Figure 3. A significant decrease in mortality was observed both for uterine cancer (AAPC = −0.6%, 95% CI = −0.8 to −0.5) and for ovarian cancer (AAPC = −0.2%, 95% CI = −0.3 to −0.1). According to the comparability test, mortality trends for ovarian cancer and for uterine cancer were not parallel (Final selected model rejected parallelism, *p* < 0.001). There were four joinpoints in the mortality trend for ovarian cancer: mortality was stable (by +0.2% per year) from 1990 to 1993, then decreased significantly from 1993 to 2013 (that is, by −0.3% per year from 1993 to 2002, and by −1.0% per year from 2002 to 2013), followed by an increase from 2013 to 2020 (that is, by +0.2% from 2012 to 2020), and then followed by an accelerated growth by +1.9% per year from 2020 until the end of the period in 2023. There were five joinpoints in the mortality trend for uterine cancer: mortality significantly decreased from 1990 to 2014 (that is, by −0.8% per year from 1990 to 2002, by −1.6% per year from 2002 to 2006, and by −0.9% per year from 2006 to 2014), followed by an insignificant increase by +0.6% per year from 2014 to 2017 and by an insignificant decrease by −0.6% per year from 2017 to 2020, and then followed by a significant increase by +1.1% per year from 2020 until the end of the period in 2023.

**Figure 4:**
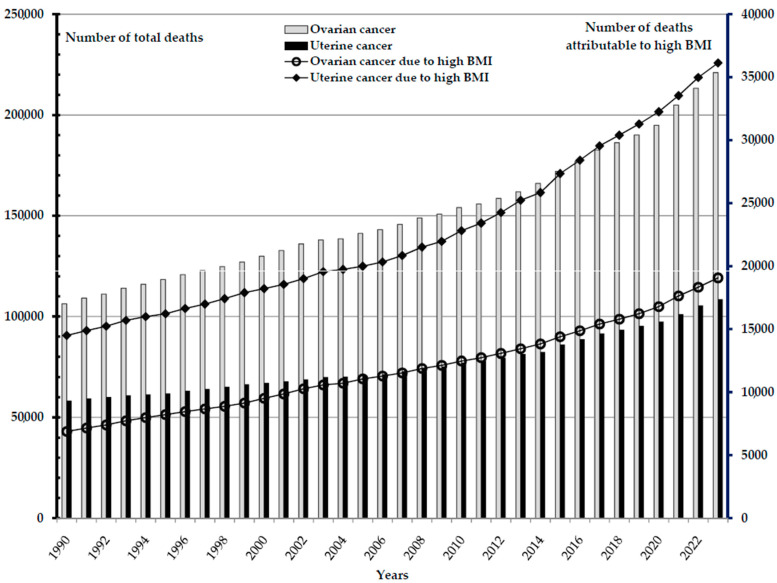
Ovarian and uterine cancer: number of total deaths and deaths attributable to high body-mass index (BMI), in the world, 1990–2023. Data source: Global Burden of Disease study [29].

A total of 1.2 million deaths from ovarian and uterine cancer attributable to high BMI were reported worldwide in the studied period, 1990–2023 (0.4 million of ovarian cancer deaths and 0.8 million of uterine cancer deaths); per year, the number of deaths ranged from 21,000 in 1990 to 55,000 in 2023 (Figure 4). In 2023, the majority of uterine cancer deaths attributable to high BMI (5677; 15.7% of the total) were recorded in the United States of America, followed by the Russian Federation and China (equally about 3000; 8.3% of the total), and then Brazil and India (equally about 1400; 3.8% of the total). Most of the deaths from ovarian cancer attributable to high BMI (2300; 12.1% of the total) were recorded in the United States of America, followed by India, China, and Russia (equally about 1300; 6.8% of the total), and then by Pakistan (850; 4.5% of the total).

**Figure 5:**
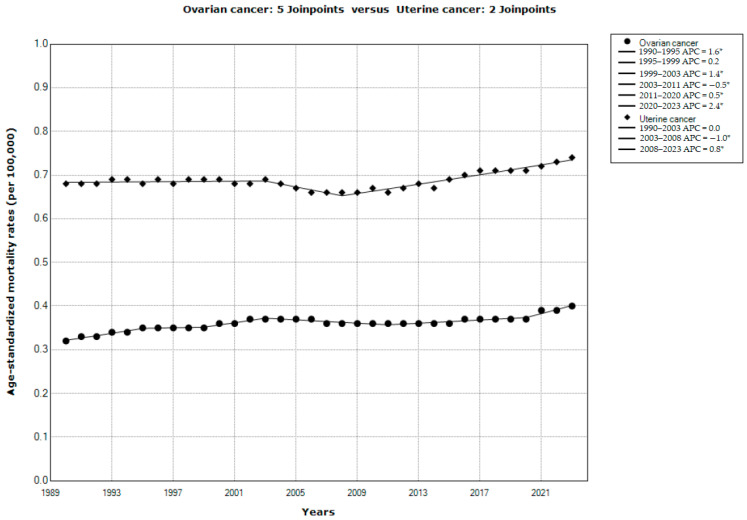
Joinpoint regression analysis of global trends in age-standardized rates of mortality from ovarian and uterine cancer attributable to high body-mass index, 1990–2023. * Statistically significant trend (*p* < 0.05). APC—annual percentage change. Data source: Global Burden of Disease study [29].

Results of the joinpoint regression analysis of the global trends in mortality from ovarian and uterine cancer attributable to high BMI in the studied period, 1990–2023, are presented in Figure 5. A significant increase in mortality was observed both for uterine cancer (AAPC = +0.2%, 95% CI = 0.1 to 0.4) and for ovarian cancer (AAPC = +0.7%, 95% CI = 0.4 to 0.9). According to the comparability test, global mortality trends for ovarian cancer and for uterine cancer attributable to high BMI were not parallel (Final selected model rejected parallelism, *p* < 0.001). There were five joinpoints in the mortality trend for ovarian cancer: mortality increased (by +1.6% per year) from 1990 to 1995, then was stable (by +0.2% per year) from 1995 to 1999, then significantly increased by +1.4% per year from 1999 to 2003, followed by a decrease by −0.5% per year from 2003 to 2011, and then significantly increased from 2011 until the end of the period in 2023 (that is, by +0.5% per year from 2011 to 2020, and with an accelerated growth by +2.4% per year from 2020 until the end of the period in 2023). There were two joinpoints in the mortality trend for uterine cancer: mortality was stable from 1990 to 2003, followed by a significant decrease by −1.0% per year from 2003 to 2008, and then significantly increased by +0.8% per year from 2008 until the end of the period in 2023.

**Figure 6:**
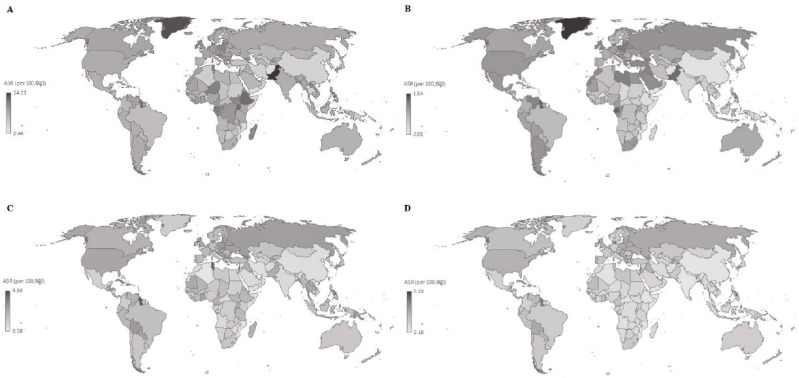
Age-standardized rates (ASRs) of mortality from ovarian and uterine cancer across 204 countries/territories in 2023: (**A**) ASR for overall ovarian cancer, (**B**) ASRs of ovarian cancer attributable to high body-mass index, (**C**) ASRs for overall uterine cancer, (**D**) ASRs of uterine cancer attributable to high body-mass index. Data source: Global Burden of Disease study [29].

Globally, the ASR of mortality from ovarian cancer was 4.6 per 100,000 in 2023 (Figure 6). The highest ASRs were found in Pakistan (14.1 per 100.000) and Ethiopia, Uganda, and Guyana (equally about 10.0 per 100.000), while the lowest rates were observed in Afghanistan, Yemen, Mozambique, Syria, Gambia, Somalia, and China (equally ≤1.0 per 100,000). Globally, the ASR of mortality from ovarian cancer attributable to high BMI was 0.4 per 100,000 in 2023; the highest ASRs attributable to high BMI were found in the Bahamas, Guyana, Gabon, Pakistan, Latvia, Trinidad and Tobago, Barbados, Bermuda, and Poland (equally about 1.2 per 100.000), while the lowest rates were observed in Somalia, Vietnam, Burkina Faso, Mozambique, Yemen, Afghanistan, and China (equally ≤0.1 per 100,000).

Globally, the ASR of mortality from uterine cancer was 2.2 per 100,000 in 2023 (Figure 6). The highest ASRs were found in Guyana, Trinidad and Tobago, and Barbados (about 7.5 per 100.000), while the lowest rates were reported in Algeria, Mozambique, Nepal, Syria, the Republic of Korea, and Iran (equally ≤1.0 per 100,000). Globally, the ASR of mortality from uterine cancer attributable to high BMI was 0.7 per 100,000 in 2023; the highest ASRs attributable to high BMI were found in Guyana, Grenada, and Barbados (equally about 1.2 per 100.000), while the lowest rates were recorded in Nepal, Vietnam, Mozambique, and Somalia (equally ≤0.2 per 100,000).

From 1990 to 2023, the global trend in ASRs of ovarian cancer deaths attributable to high BMI significantly increased (AAPC = +0.4%, 95% CI = 0.3 to 0.5) (Table 1). The growth trend in South Asia (AAPC = +8.7%, 95% CI = 8.1 to 9.2) was 30 times faster than in Eastern Europe (AAPC = +0.3%, 95% CI = 0.1 to 0.5). Declining trends in mortality from ovarian cancer attributable to high BMI were observed only in Australasia (AAPC = −0.2%, 95% CI = −0.4 to −0.1), High-income North America (AAPC = −0.3%, 95% CI = −0.6 to −0.0), and Western Europe (AAPC = −0.7%, 95% CI = −0.8 to −0.6). The global trend in mortality from uterine cancer attributable to high BMI significantly increased (AAPC = +0.1, 95% CI = 0.0 to 0.2), with the trend growing fastest in South Asia (AAPC = +4.2%, 95% CI = 4.0 to 4.4). Declining trends in mortality from uterine cancer attributable to high BMI were observed only in Central Asia (AAPC = −0.6%, 95% CI = −0.9 to −0.4), East Asia (AAPC = −2.2%, 95% CI = −2.6 to −1.8), and Southern Latin America (AAPC = −0.4%, 95% CI = −0.6 to −0.1).

**Figure 7:**
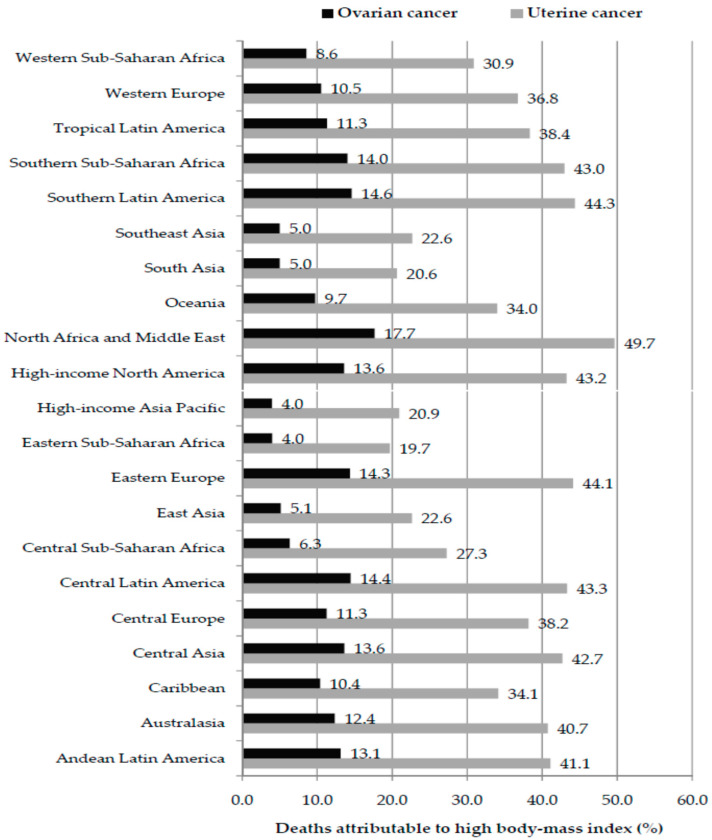
Fraction (%) of deaths from ovarian and uterine cancer attributable to high body-mass index by GBD region, in 2023. Data source: Global Burden of Disease study [29].

Globally, the fraction of deaths from ovarian cancer attributable to high BMI in 2023 was 8.65%, while for uterine cancer it was 33.33% (Figure 7). In both ovarian and uterine cancer, fractions of deaths attributable to high BMI were the highest in the GBD region of North Africa and Middle East (17.7% and 49.7%, respectively). Also, in both ovarian and uterine cancer, fractions of deaths attributable to high BMI were the lowest in the GBD region of Eastern Sub-Saharan Africa (4.0% and 19.7%, respectively). Large disparities (five-fold) in the fraction of ovarian cancer deaths attributable to high BMI were observed between the regions with the highest fraction (North Africa and Middle East, Latin America, Eastern Europe, and High-income North America) and regions with the lowest fraction (High-income Asia Pacific, Eastern Sub-Saharan Africa, East Asia, South Asia, and Southeast Asia). Disparities (two-fold) in the fraction of uterine cancer deaths attributable to high BMI were observed between the regions with the highest fraction (North Africa and Middle East, Latin America, Eastern Europe, and High-income North America) and regions with the lowest fraction (High-income Asia Pacific, East Asia, South Asia, Southeast Asia, and Eastern Sub-Saharan Africa).

**Figure 8:**
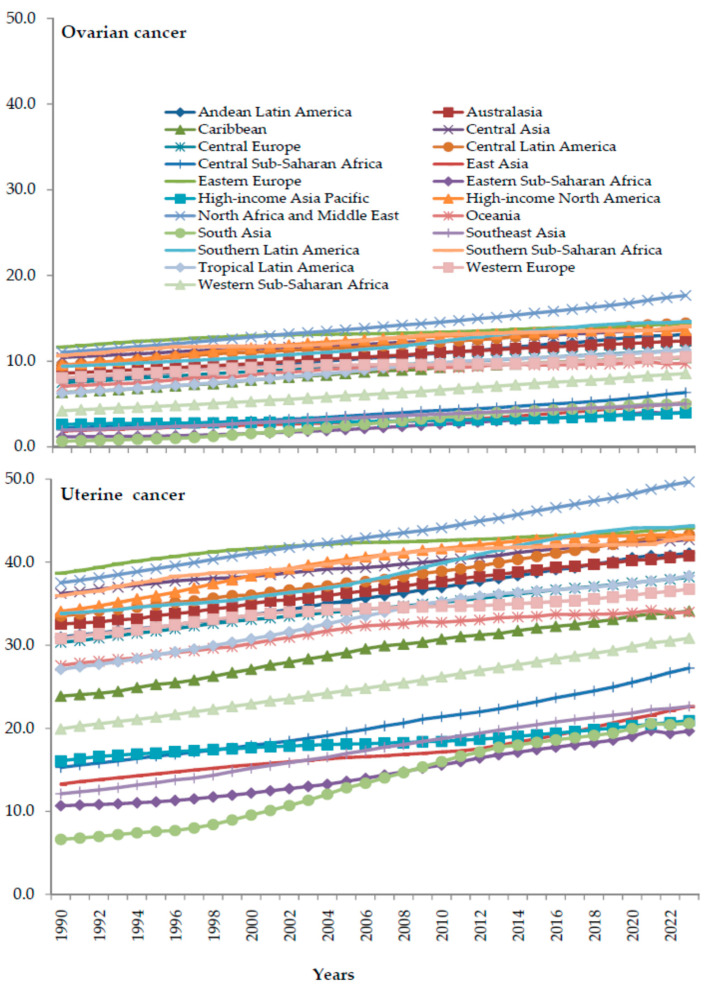
Fraction (%) of deaths from ovarian and uterine cancer attributable to high body-mass index by GBD region, in 1990–2023. Data source: Global Burden of Disease study [29].

In both ovarian and uterine cancer, fractions (%) of deaths attributable to high BMI increased across all GBD regions from 1990 to 2023 (Figure 8). In both ovarian and uterine cancer, an increase in the fraction of deaths attributable to high BMI was greatest in the GBD region of Central Asia, especially from the 2000s to 2023.

Globally, across all age groups apart from the elderly (65–89 years), the ovarian cancer mortality rates attributable to high BMI showed a significant (*p* < 0.001) upward trend over the observed period 1990–2023, with the most rapid increase observed in the youngest age group (20–29 years) (Table 2). The mortality increased with age, and estimates for each age group were higher in 2023 than in 1990. The highest rates were observed in the 75–95-year age group in both 1990 and 2023.

Age-specific mortality rates for uterine cancer attributable to high BMI increased with age and were higher in all age groups in 2023 compared with 1990. The highest age-specific rates were observed in persons older than 75 years, with significantly upward trends, and the most rapid increase occurred in the oldest age group (95+ years). Globally, across all age groups below 75 years, the trends in age-specific mortality rates for uterine cancer attributable to high BMI remained stable from 1990 to 2023 (*p* > 0.05).

**Figure 9:**
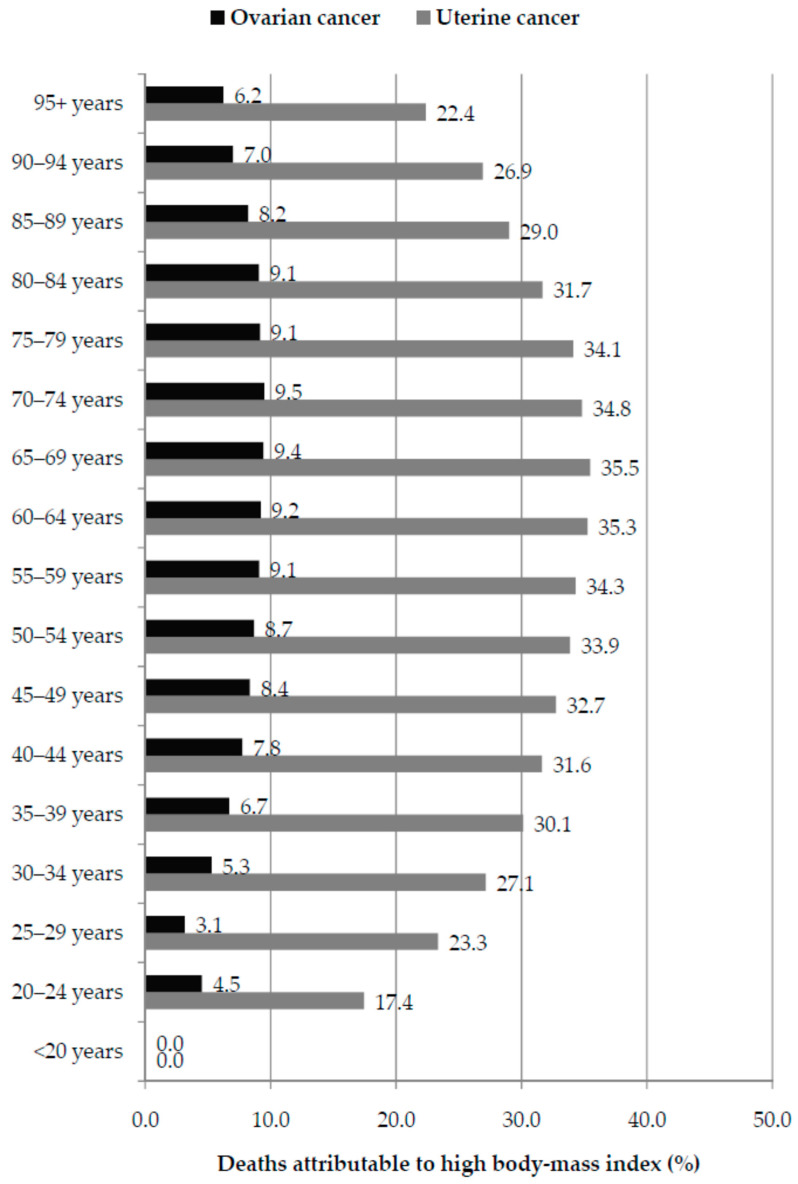
Global fraction (%) of deaths of ovarian and uterine cancer attributable to high body-mass index by age group, in 2023. Data source: Global Burden of Disease study [29].

For uterine cancer, global fractions of deaths attributable to high BMI were the highest in those aged 60–64 and 65–69 (35.3% and 35.5%, respectively) (Figure 9). For ovarian cancer, global fractions of deaths attributable to high BMI were the highest in those aged 65–69 and 70–74 (9.4% and 9.5%, respectively) (Figure 9). In both ovarian and uterine cancer, fractions of deaths attributable to high BMI were the lowest in the youngest and oldest age groups.

**Figure 10:**
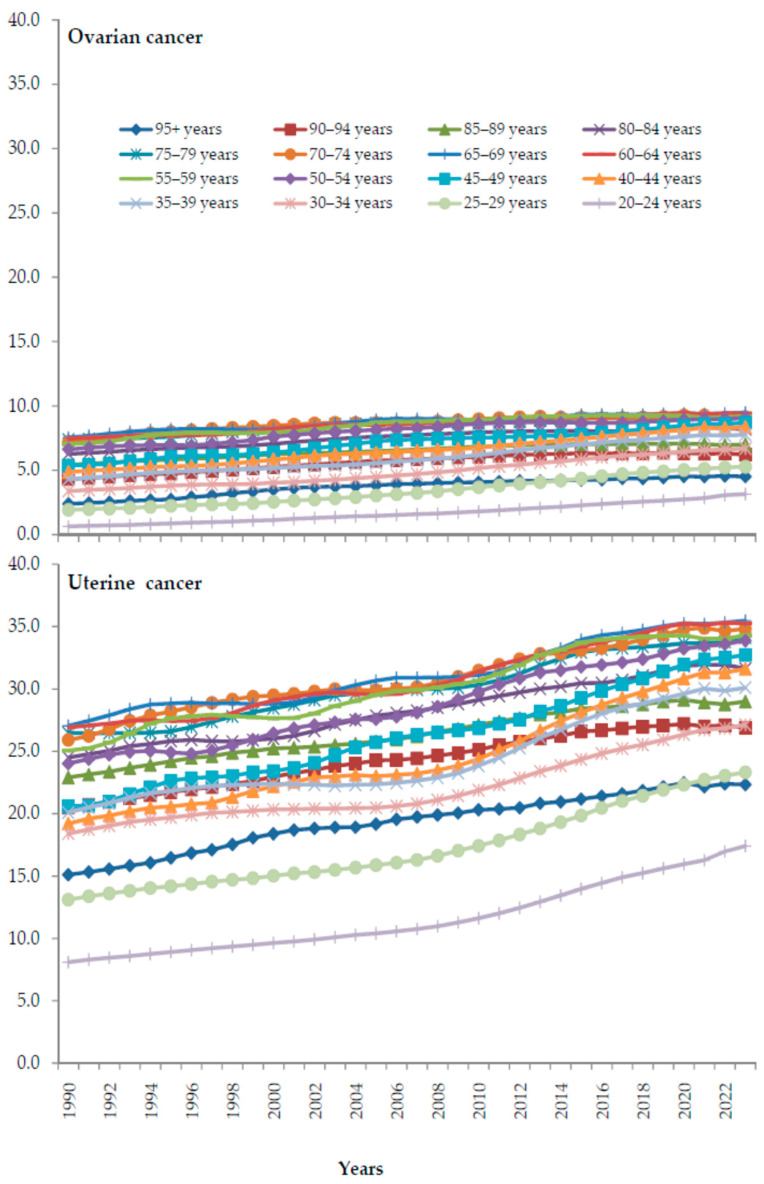
The global trends in fractions (%) of ovarian and uterine cancer deaths attributable to high body-mass index, by age group, from 1990 to 2023. Data source: Global Burden of Disease study [29].

In both ovarian and uterine cancer, fractions (%) of deaths attributable to high BMI increased in all age groups (20 and over) from 1990 to 2023 (Figure 10). In both ovarian and uterine cancer, an increase in the fraction of deaths attributable to high BMI was the fastest in age groups spanning 65–79 years.

**Figure 11:**
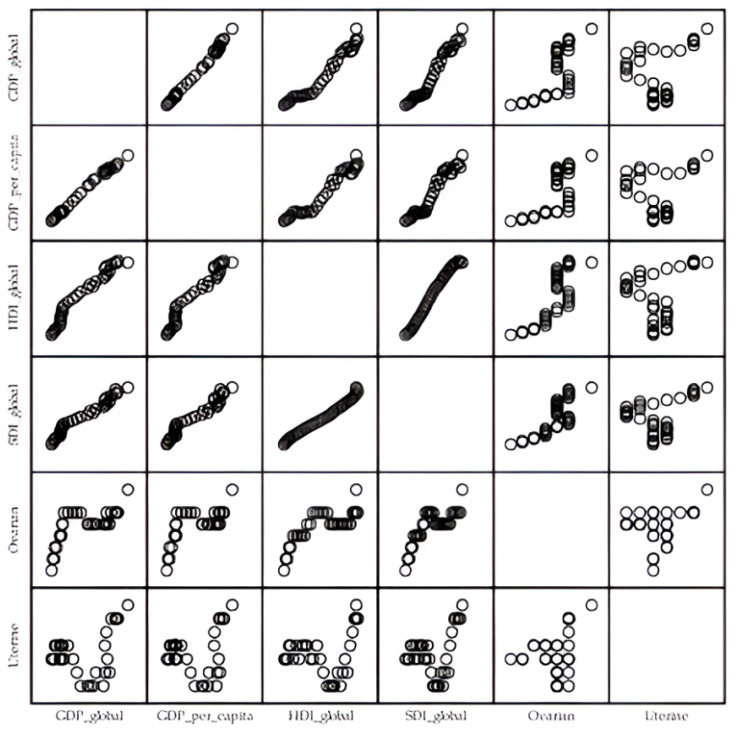
Association of the global age-standardized mortality rates (ASMR, per 100,000) for ovarian and uterine cancer attributable to high body-mass index with global Gross Domestic Product (GDP), GDP per capita, Human Development Index (HDI), and Sociodemographic Index (SDI), in 1990–2023. Data sources: Global Burden of Disease study [29] and United Nations [34].

Linear regression analyses showed a significant positive association between the global age-standardized mortality rates (per 100,000) for ovarian cancer attributable to high BMI and global GDP, GDP per capita, HDI, and SDI in 1990–2023 (R2 = 0.595, *p* = 0.000; R2 = 0.569, *p* = 0.000; R2 = 0.656, *p* = 0.000; R2 = 0.694, *p* = 0.000, respectively) (Figure 11). Linear regression analyses showed a significant positive association between the global age-standardized mortality rates (per 100,000) for uterine cancer attributable to high BMI and global GDP, GDP per capita, HDI, and SDI in 1990–2023 (R2 = 0.231, *p* = 0.004; R2 = 0.173, *p* = 0.014; R2 = 0.157, *p* = 0.020; R2 = 0.245, *p* = 0.003, respectively).

**Figure 12:**
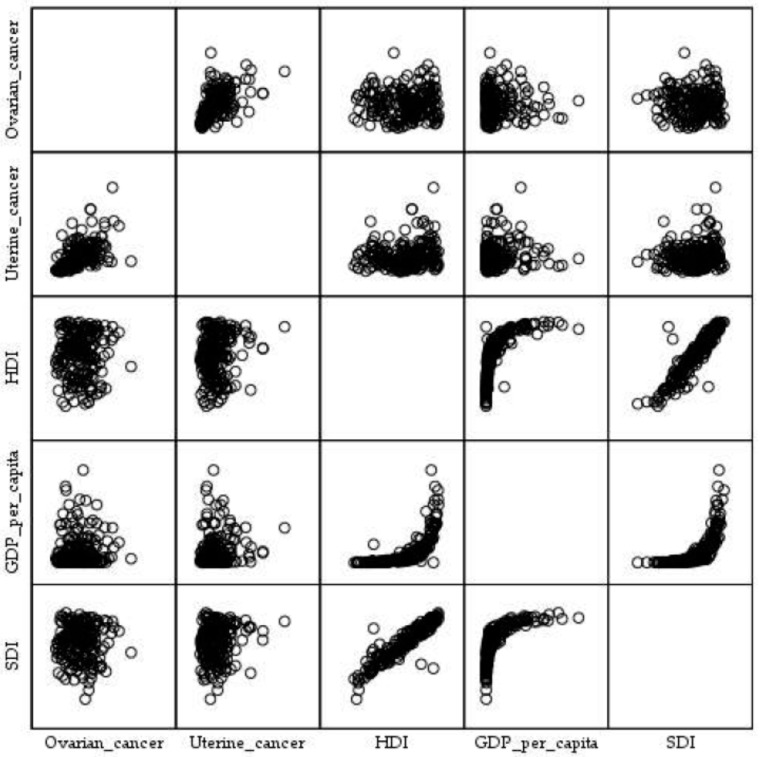
Correlation of the age-standardized mortality rates (ASMR, per 100,000) for ovarian and uterine cancer attributable to high body-mass index with Gross Domestic Product per capita (GDP per capita), Human Development Index (HDI), and Sociodemographic Index (SDI), by country in 2023. Data sources: Global Burden of Disease study [29] and United Nations [34].

The Pearson correlation coefficient showed an absence of correlation between the age-standardized mortality rates (per 100,000) for ovarian cancer attributable to high BMI and HDI (r = 0.019; *p* = 0.795), GDP per capita (r = 0.009; *p* = 0.900), and SDI (r = 0.005; *p* = 0.940), by countries in 2023 (Figure 12).

The Pearson correlation coefficient showed an absence of correlation between the age-standardized mortality rates (per 100,000) for uterine cancer attributable to high BMI and GDP per capita (r = 0.085; *p* = 0.240), while a positive correlation was observed for HDI (r = 0.151; *p* = 0.037) and SDI (r = 0.158; *p* = 0.027), by countries in 2023.

## 4. Discussion

Marked discrepancies were observed in patterns and temporal trends of mortality from ovarian and uterine cancer attributable to high BMI worldwide. Notably, increasing trends in the burden of both ovarian and uterine cancer worldwide particularly accelerated during 2020–2023, corresponding to the COVID-19 pandemic period.

Apparent geographic differences in patterns and trends of mortality from ovarian and uterine cancer attributable to high BMI could be explained by different age structures across populations, demographic growth, variations in life expectancy, availability of healthcare services, improvements in cancer management, preventive measures, and practices of cancer certification and registration [2,5]. The notion that an increase in global burden of ovarian and uterine cancer may reflect aging and growth of the world population is supported by the results of the GBD 2021 study, which reported the proportion of people aged ≥70 being 6.3% of the total world population of 7.9 billion in 2021, up from 3.8% of the 5.3 billion in 1990 [29]. Apart from genetic factors, the burden of ovarian and uterine cancer could reflect many other factors like the frequency of autopsies, representation and heterogeneity of histological subtypes, certain environmental risks, obesity, etc. [41,42].

Obesity is considered a risk factor for ovarian [43] and uterine cancer [20]. Also, research shows that high BMI could have an effect on mortality from these cancers, but the evidence is heterogenous, which could explain noted discrepancies in ovarian and uterine cancer mortality attributable to high BMI worldwide. Studies have indicated a survival disadvantage in patients with ovarian cancer and high BMI [44], but results are inconsistent [45,46,47,48]. For uterine cancer, one evidence synthesis showed that high BMI was significantly associated with higher all-cause mortality [49], while others showed no impact on cancer-specific mortality [50,51]. For ovarian cancer and obesity, a large meta-analysis found no difference in overall or cancer-specific survival [51], while pooled data from 21 case–control studies showed disadvantage in overall survival and cancer-specific mortality [52].

Tumor histology could also explain the heterogenous evidence. One study found a significant association between ovarian cancer survival and high BMI only for the high-grade serous subtype [52]. A large study of global surveillance of cancer survival in 51 countries (CONCORD-2) reported that high-grade serous carcinoma made up over 70% of ovarian tumors in Oceania, North America, and Europe, compared to 66% and 56% in South America and Asia, respectively [53]. Other subtypes were registered more frequently in Asia than in North America (32.5% vs. 19.4%). Similar results were noted in a study of data from registries of the Cancer Incidence in Five Continents project [54]. For uterine cancer, increased mortality associated with high BMI was noted for both histological type I (the most common worldwide) and type II [55], but results were heterogenous [56]. The faster increase in uterine cancer mortality attributable to high BMI in South Asia may be associated with lifestyle changes due to westernization, rising obesity prevalence, changes in the age of menarche and menopause, and metabolic and hormonal changes, all of which can contribute both to the onset and progression of disease [57]. Contributing factors could also relate to healthcare access, surveillance of high-risk populations, and surgery wait time [58]. Regions with a decrease in ASRs of mortality from uterine cancer attributable to high BMI could have experienced improvements in diagnosis and treatment, with the introduction of clinical guidelines and new treatment modalities [58,59]. Still, predictions of mortality trends from uterine cancer attributable to metabolic risk factors showed that, despite declines in some regions, a stagnation in decline was noted, which highlights the need for timely action and continued preventive efforts for metabolic risk factors and early detection [60]. Also, the predicted decline in total fertility rate across these regions by 2050 and onward [61], changes in reproductive factors and their interplay with metabolic factors, along with the later onset of menstruation and earlier menopause [62], all point to the need for improved and continued disease monitoring.

Another possible explanation for the observed discrepancies could be the stage of disease. Namely, research suggests that obesity is linked with significantly worse ovarian cancer survival for localized disease (stages I/II), contrary to late-stage (IV) [45], while in endometrial cancer, high BMI was associated with an increased risk of death both in early stage and advanced disease (stages III and IV) [49,63]. Our findings of a significant positive association between the age-standardized mortality rates from ovarian cancer attributable to high BMI and GDP, GDP per capita, HDI, and SDI and findings that South Asia and Eastern Sub-Saharan Africa regions experienced the highest increase in ASR of mortality attributable to high BMI could be interpreted in the context of countries with higher level of development being associated with higher prevalence of obesity and better data quality. Furthermore, the increasing mortality attributable to high BMI in some lower-resource settings could possibly reflect delays in diagnosis; the complexity of treatment of a patient with cancer and obesity, which involves risks related to surgical treatment and chemotherapy dosages; and risks related to comorbidities [64,65]. Thus, some factors could be responsible for the observed differences in mortality attributable to high BMI regardless of predominant cancer subtypes in certain locations, but impairing survival via health-system weaknesses, including challenges in making a diagnosis and consequently diagnoses at late stages [66]. Nevertheless, the observed correlations between socioeconomic indicators and cancer mortality attributable to high BMI should be interpreted in the context of the applied ecological study design and further elucidated in analytical studies.

Research has shown different effects of obesity at early adulthood and in years prior to diagnosis, compared to obesity at diagnosis [50,67]. A meta-analysis found significantly lower survival in women with ovarian cancer who were obese in early adulthood and those obese 5 years prior to diagnosis [67]. Contrary to this, results of a large prospective study of post-menopausal women enrolled in the Women’s Health Initiative Study found that BMI from an average of 7 years prior to diagnosis was not associated with mortality [68]. For uterine cancer, research mostly indicates a significant positive association between mortality and high BMI measured ≥5 years prior to diagnosis [69,70,71], but there is always the question of distinguishing the association with incidence from that with mortality [72]. Pre-clinical research shows that obesity is associated with enhanced metastatic potential, poor response to treatment, and worse survival [73]. Thus, a longer duration of such metabolic influence on the tumor’s microenvironment could serve as a promoter of the tumor’s aggressiveness [74,75]. Notably, many of the locations with the highest ASR of mortality from ovarian and uterine cancer attributable to high BMI observed in this study experienced an increase in obesity prevalence over the 1990–2022 period or have had their double burden of underweight and obesity shift towards obesity dominance toward the end of this period [76]. The highest increase in ovarian and uterine cancer mortality attributable to high BMI was observed in South Asia, the region that has seen the sharpest increase in mortality from cancers attributable to high BMI [77]. This region is undergoing a transition from undernutrition to obesity due to the adoption of a Western lifestyle, a diet rich in sugar and fat, urbanization, and less physical activity [76,78], which might lead to women being obese at younger ages and a longer duration of obesity. Contrary to that, in Australasia, high-income North America, and Western Europe, a significant decrease in mortality from ovarian cancer attributable to high BMI was observed; these patterns may reflect established interventions for obesity control, as well as a more developed healthcare system. Nevertheless, obesity has mostly been increasing globally over the last decades, or at least remained high [76], thus the existing efforts aimed at obesity prevention and control should be continued and strengthened. The present study showed a significant increase in global trends in mortality from ovarian cancer attributable to high BMI across all age groups < 65 years, with the highest increases in younger age groups, while for uterine cancer decreases in ASRs of mortality attributable to high BMI were noted in regions which experienced improvements in early detection, diagnostics, in younger population’s disease awareness, and different socioeconomic and lifestyle changes [79,80,81].

For both ovarian and uterine cancer, a notable increase in deaths attributable to high BMI was observed towards the end of the considered period, which corresponds to the COVID-19 pandemic. Research has shown substantial disruptions in healthcare systems worldwide during the pandemic, including a significant reduction in screening, delays in diagnoses, reduced access to treatment, and a decline in treatment completion. The regular screening processes were disrupted, leading to delays in cancer diagnosis [82]. Many surgical and other treatment procedures suffered delays due to healthcare systems being redirected and strained with COVID-19 management [83]. Research indicates that longer wait times until treatment negatively impacted survival [84], with >6 weeks between the diagnosis and surgery for epithelial endometrial cancer being associated with significantly lower survival [85], and a waiting time of ≥4 weeks being associated with a significant decrease in survival for ovarian cancer. [86]. Additionally, surgical procedures in SARS-CoV-2 positive patients were recommended for a delay of at least 7 weeks after the infection [87]. According to a large international prospective cohort study, the pandemic lockdowns have caused almost three-quarters of surgical procedures for gynecological cancers to take 5 or more weeks from diagnosis to surgery [83]. The COVID-19 pandemic influenced healthcare delivery, cancer detection, and treatment, all of which represent a context that could plausibly influence mortality patterns. Whether or not the observed increase in ovarian and uterine cancer mortality attributable to high BMI during the pandemic period was pandemic-driven needs to be determined in future studies.

Ovarian cancer has a poor 5-year survival rate of 30–50% [88,89], with over half of new cases being diagnosed at a very advanced stage and about the same amount experiencing disease recurrence [90]. For uterine cancer, the 5-year survival rate is around 80% [1,2,4]. Thus, monitoring the trends of factors that could be associated with survival is crucial for targeted evidence-based preventive strategies. Global disparities in mortality from ovarian an uterine cancer attributable to high BMI should be considered in the context of possible explanations which could relate to the quality of data, presence of contributing comorbidities, means of BMI measurement (self-reported or verified), differences in BMI classification over time and the appropriateness of BMI as a measurement of visceral fat which would reflect the microenvironment that influences cancer development, progression and prognosis [91]. Some decreases in gynecologic cancer mortality rates attributable to high BMI seen in regions with high obesity prevalence could be explained with the obesity paradox phenomenon, where obese persons with cancer experience a survival improvement; despite some heterogenous findings at the individual study level [92,93], a recent meta-analysis specific to gynecologic cancers did not confirm a positive effect, and found a significant decrease of 44% in the survival of endometrial cancer in women with a high BMI [94]. Also, over the 1990–2023 period, a global increase in deaths attributable to high BMI for both ovarian and uterine cancer was the highest in age groups spanning 65–79 years, possibly related to comorbidities and longer duration of obesity in older women. This, alongside global population aging, underlines the need for enforcing policies that will improve obesity prevention and control, promote healthy eating habits, reduce the consumption of ultra-processed food, increase physical activity, and improve women’s awareness of ovarian and uterine cancer. Further research is needed to clarify the extent to which the accelerated growth in mortality from ovarian and uterine cancer attributable to high BMI observed at the end of the considered period may reflect the disruptions in healthcare systems during the COVID-19 pandemic or ongoing underlying trends. Also, the observed accelerated upward trends in ovarian and uterine cancer mortality attributable to high BMI in the 2020–2023 period, which corresponds to the period of the COVID-19 pandemic, highlight the importance of timely assessment and monitoring of long-term population health trends.

### Strengths and Limitations of the Research

This study analyzed global patterns and temporal trends of mortality for ovarian and uterine cancer from 1990 to 2023, as well as the contribution of high BMI to mortality worldwide. Also, this study assessed the association of the mortality from ovarian and uterine cancer attributable to high BMI with indicators of human development in the world. In addition, joinpoint regression analysis allowed for the precise determination and interpretation of the direction and magnitude of changes in mortality trends over time. Finally, this study used data from the GBD 2023 database, which provides comprehensive, high-quality, and up-to-date mortality information for the entire world.

However, some limitations of this study should be considered. First, questions inevitably arise regarding the reliability, accuracy, and coverage of mortality statistics for ovarian and uterine cancer attributable to high BMI. Namely, regional variations in data quality may persist, reflecting varying capacities for collecting, registering, and reporting cancer mortality data across different levels of socio-economic development. Potential sources of bias in estimating trends in ovarian and uterine cancer mortality attributable to high BMI worldwide may include inconsistent availability and variable quality of information on prevalence of high BMI collected from some self-reported data (which may result in an underestimation of BMI) or obtained from samples not representative of the entire country, especially in developing or low-income countries where data on BMI are sometimes sparse. In addition, it should be noted that BMI does not necessarily reflect body composition and adiposity distribution. Furthermore, this study did not examine some potential covariates such as genetics, hormone therapy, reproductive history, personal medical history, diabetes mellitus, alcohol use, etc. Furthermore, stratification across different histological subtypes of ovarian and uterine cancer was not currently available within the GBD study, and future analyses with such data would enable a more granular assessment of epidemiological patterns. Finally, the epidemiological fallacy inherent in the design of an ecological study poses a large problem for this research. Consequently, the findings of this study regarding the correlation between the mortality from ovarian and uterine cancer and high BMI must be further evaluated in analytical epidemiological studies.

## 5. Conclusions

From 1990 to 2023, global trends in mortality from both ovarian and uterine cancer attributable to high BMI increased significantly, with accelerating growth observed during the 2020–2023 period, which corresponds to the COVID-19 pandemic. Almost all regions experienced unfavorable trends in mortality from ovarian and uterine cancer attributable to high BMI, with the increase being the highest in South Asia. Declining trends in mortality rates for ovarian cancer attributable to high BMI were observed only in the most developed areas, such as the regions of Australasia, High-income North America, and Western Europe, while for uterine cancer, only in developing areas, such as the regions of Central Asia, East Asia, and Southern Latin America. Mortality rates for both ovarian and uterine cancer attributable to high BMI increased with age and were higher in every age group in 2023 than in 1990. These findings highlight the significance of public health strategies for the prevention and management of ovarian and uterine cancer, particularly through programs of obesity prevention and control. Further analytical epidemiological studies are needed to clarify the relationship between high BMI and ovarian and uterine cancer.

## Figures and Tables

**Table 1:** Mortality trends of ovarian and uterine cancer attributable to high body mass index, by location, 1990–2023; a joinpoint regression analysis.

	Ovarian Cancer			Uterine Cancer		
Locations	Count in 2023	ASR in 2023	AAPC (ASR) (95% CI)	Count in 2023	ASR in 2023	AAPC (ASR) (95% CI)
Global	19,069	0.40	0.4 * (0.3 to 0.5)	36,136	0.74	0.1 * (0.0 to 0.2)
GBD regions						
Andean Latin America	209	0.61	4.2 * (3.6 to 4.8)	456	1.33	0.0 (−0.3 to 0.3)
Australasia	178	0.59	−0.2 * (−0.4 to −0.1)	311	1.00	0.9 * (0.7 to 1.1)
Caribbean	139	0.47	2.2 * (2.1 to 2.4)	553	1.85	1.9 * (1.7 to 2.1)
Central Asia	251	0.48	1.1 * (0.9 to 1.3)	539	1.05	−0.6 * (−0.9 to −0.4)
Central Europe	939	0.75	1.7 * (1.4 to 1.9)	2023	1.50	0.6 * (0.4 to 0.7)
Central Latin America	993	0.67	2.3 * (2.1 to 2.4)	1365	0.93	1.1 * (0.7 to 1.5)
Central Sub-Saharan Africa	163	0.40	5.6 * (5.1 to 6.1)	169	0.45	3.4 * (3.1 to 3.7)
East Asia	1295	0.11	1.2 * (0.7 to 1.6)	3115	0.26	−2.2 * (−2.6 to −1.8)
Eastern Europe	1613	0.76	0.3 * (0.1 to 0.5)	4283	1.88	0.3 * (0.1 to 0.5)
Eastern Sub-Saharan Africa	349	0.27	6.1 * (5.9 to 6.4)	547	0.48	3.1 * (2.9 to 3.3)
High-income Asia Pacific	316	0.15	0.9 * (0.7 to 1.1)	985	0.43	1.0 * (0.7 to 1.2)
High-income North America	2568	0.73	−0.3 * (−0.6 to −0.0)	6215	1.69	1.7 * (1.6 to 1.9)
North Africa and Middle East	1585	0.62	2.3 * (2.2 to 2.4)	1973	0.81	1.1 * (1.0 to 1.2)
Oceania	9	0.19	2.5 * (2.3 to 2.7)	62	1.43	1.4 * (1.3 to 1.5)
South Asia	2455	0.29	8.7 * (8.1 to 9.2)	2397	0.29	4.2 * (4.0 to 4.4)
Southeast Asia	985	0.24	4.6 * (4.4 to 4.8)	1976	0.49	2.8 * (2.7 to 2.8)
Southern Latin America	373	0.74	1.0 * (0.9 to 1.1)	541	1.01	−0.4 * (−0.6 to −0.1)
Southern Sub-Saharan Africa	264	0.65	2.2 * (1.9 to 2.5)	374	0.96	1.7 * (1.4 to 2.0)
Tropical Latin America	636	0.43	1.9 * (1.7 to 2.1)	1484	0.99	0.6 * (0.5 to 0.7)
Western Europe	3131	0.61	−0.7 * (−0.8 to −0.6)	5884	1.06	0.7 * (0.6 to 0.8)
Western Sub-Saharan Africa	620	0.45	3.7 * (3.4 to 3.9)	883	0.74	2.1 * (1.8 to 2.3)

ASR—age-standardized rates (per 100,000). For full period (1990–2023) presented, AAPC—average annual percentage change; 95% CI—confidence interval; GBD—Global Burden of Disease. * Statistically significant trend (*p* < 0.05). Data source: Global Burden of Disease study [29].

**Table 2:** Global mortality from ovarian and uterine cancer attributable to high body mass index, by age group, 1990–2023; a joinpoint regression analysis.

Age (Years) **	1990		2023		1990–2023	
	Count	Rate (per 100,000)	Count	Rate (per 100,000)	AAPC (%)	95% CI
	Ovarian cancer					
20–24	7	0.00	53	0.02	5.0 *	4.7 to 5.3
25–29	24	0.01	130	0.04	4.0 *	3.8 to 4.3
30–34	60	0.03	282	0.10	3.0 *	2.7 to 3.4
35–39	115	0.07	449	0.16	2.3 *	1.9 to 2.6
40–44	219	0.16	790	0.31	1.6 *	1.3 to 2.0
45–49	360	0.32	1273	0.54	1.1 *	0.9 to 1.3
50–54	670	0.64	1897	0.84	0.6 *	0.5 to 0.7
55–59	830	0.90	2274	1.08	0.4 *	0.3 to 0.5
60–64	1069	1.30	2587	1.48	0.3 *	0.2 to 0.4
65–69	1104	1.66	2686	1.79	0.0	0.0 to 0.0
70–74	852	1.81	2377	2.00	−0.1	−0.2 to 0.1
75–79	808	2.21	1829	2.30	−0.0	−0.2 to 0.1
80–84	485	2.16	1308	2.45	0.2	−0.0 to 0.4
85–89	214	2.06	738	2.34	0.2	−0.0 to 0.5
90–94	61	1.93	313	2.36	0.7 *	0.4 to 1.0
95+	9	1.09	81	1.82	1.6 *	1.3 to 2.0
Total/Age-standardized:	6887	0.32	19,069	0.40	0.4 *	0.3 to 0.5
	Uterine cancer					
20–24	25	0.01	33	0.01	−0.1	−0.5 to 0.3
25–29	60	0.03	106	0.04	0.2	−0.2 to 0.5
30–34	130	0.07	246	0.08	−0.2	−0.5 to 0.1
35–39	239	0.14	430	0.15	−0.3	−0.5 to 0.0
40–44	341	0.24	771	0.30	0.1	−0.1 to 0.4
45–49	536	0.47	1358	0.58	0.2	−0.0 to 0.3
50–54	1019	0.97	2309	1.02	0.0	−0.1 to 0.1
55–59	1446	1.57	3348	1.60	−0.0	−0.1 to 0.1
60–64	2274	2.77	5062	2.89	0.1	−0.1 to 0.3
65–69	2485	3.74	5925	3.95	0.0	−0.1 to 0.2
70–74	1998	4.25	5658	4.77	0.1	−0.1 to 0.2
75–79	1898	5.19	4335	5.44	0.2 *	0.0 to 0.3
80–84	1232	5.49	3238	6.06	0.2 *	0.2 to 0.3
85–89	573	5.53	2018	6.41	0.4 *	0.3 to 0.5
90–94	197	6.23	980	7.38	0.7 *	0.6 to 0.8
95+	43	5.34	318	7.11	1.1 *	1.0 to 1.2
Total/Age-standardized:	14,495	0.68	36,136	0.74	0.1 *	0.0 to 0.2

For full period (1990–2023) presented, AAPC—average annual percentage change; 95% CI—confidence interval. * Statistically significant trend (*p* < 0.05). ** Results are not shown for mortality in persons aged <20, because no case of death of ovarian and uterine cancer attributable to high body-mass index occurred in at least one year in the observed period. Data source: Global Burden of Disease study [29].

## Data Availability

Data is contained within the article.
